# Examining the potential benefits of the influenza vaccine against SARS-CoV-2: A retrospective cohort analysis of 74,754 patients

**DOI:** 10.1371/journal.pone.0255541

**Published:** 2021-08-03

**Authors:** Susan M. Taghioff, Benjamin R. Slavin, Tripp Holton, Devinder Singh

**Affiliations:** 1 Division of Plastic & Reconstructive Surgery, University of Miami Miller School of Medicine, Miami, Florida, United States of America; 2 Anne Arundel Medical Center, Annapolis, Maryland, United States of America; Erasmus Medical Centre: Erasmus MC, NETHERLANDS

## Abstract

**Introduction:**

Recently, several single center studies have suggested a protective effect of the influenza vaccine against severe acute respiratory syndrome coronavirus-2 (SARS-CoV-2). This study utilizes a continuously updated Electronic Medical Record (EMR) network to assess the possible benefits of influenza vaccination mitigating critical adverse outcomes in SARS-CoV-2 positive patients from 56 healthcare organizations (HCOs).

**Methods:**

The de-identified records of 73,346,583 patients were retrospectively screened. Two cohorts of 37,377 patients, having either received or not received influenza vaccination six months–two weeks prior to SARS-CoV-2 positive diagnosis, were created using Common Procedural Terminology (CPT) and logical observation identifiers names and codes (LOINC) codes. Adverse outcomes within 30, 60, 90, and 120 days of positive SARS-CoV-2 diagnosis were compared between cohorts. Outcomes were assessed with stringent propensity score matching including age, race, ethnicity, gender, hypertension, diabetes, hyperlipidemia, chronic obstructive pulmonary disease (COPD), obesity, heart disease, and lifestyle habits such as smoking.

**Results:**

SARS-CoV-2-positive patients who received the influenza vaccine experienced decreased sepsis (p<0.01, Risk Ratio: 1.361–1.450, 95% CI:1.123–1.699, NNT:286) and stroke (p<0.02, RR: 1.451–1.580, 95% CI:1.075–2.034, NNT:625) across all time points. ICU admissions were lower in SARS-CoV-2-positive patients receiving the influenza vaccine at 30, 90, and 120 days (p<0.03, RR: 1.174–1.200, 95% CI:1.003–1.385, NNT:435), while approaching significance at 60 days (p = 0.0509, RR: 1.156, 95% CI:0.999–1.338). Patients who received the influenza vaccine experienced fewer DVTs 60–120 days after positive SARS-CoV-2 diagnosis (p<0.02, RR:1.41–1.530, 95% CI:1.082–2.076, NNT:1000) and experienced fewer emergency department (ED) visits 90–120 days post SARS-CoV-2-positive diagnosis (p<0.01, RR:1.204–1.580, 95% CI: 1.050–1.476, NNT:176).

**Conclusion:**

Our analysis outlines the potential protective effect of influenza vaccination in SARS-CoV-2-positive patients against adverse outcomes within 30, 60, 90, and 120 days of a positive diagnosis. Significant findings favoring influenza vaccination mitigating the risks of sepsis, stroke, deep vein thrombosis (DVT), emergency department (ED) & Intensive Care Unit (ICU) admissions suggest a potential protective effect that could benefit populations without readily available access to SARS-CoV-2 vaccination. Thus further investigation with future prospective studies is warranted.

## Introduction

With cases in excess of 140 million and a death toll over 3 million, COVID-19 has greatly impacted the global community [1]. In the nascency of severe acute respiratory syndrome coronavirus-2 (SARS-CoV-2), demand for rapid, yet accurate data was voracious [2]. As the world continues to attempt to overcome the current pandemic and readies itself to combat a future one, the need for expeditious clinical answers remains paramount.

Federated electronic medical record (EMR) networks, such as TriNetX (TriNetX Inc, Cambridge, MA), aggregate the de-identified records of millions of patients from participating healthcare organizations (HCOs) into an accessible and searchable database in real-time [3, 4]. Several publications have already demonstrated the utility of federated EMR networks in addressing research questions regarding the implications of SARS-CoV-2 on maladies including obesity, rheumatological disease, gastrointestinal bleeding, and psychiatric illness [5–8]. The efficiency and speed with which these previous retrospective studies were able to examine topics of interest, using real-time EMRs, allows for the collective advancement of COVID-19 knowledge in hopes of optimizing prevention and management.

Recently, several studies have suggested a possible protective effect of the influenza vaccine against SARS-CoV-2 [9–12]. Although no cross-reactivity between influenza-induced antibodies and SARS-CoV-2 protection has been demonstrated, several theorized mechanisms of the potential protective effect of influenza vaccination have been proposed in the recent literature [9, 13, 14]. The first hypothesis centers around the presence of MF59 in the influenza vaccine: an oil-in-water squalene emulsion that has been shown to assist in potentiating an immune response to SARS-CoV variants [14]. Alternatively, influenza vaccination’s potential protective effect may be explained by its ability to stimulate the activation of natural killer cells, the levels of which have been found to be considerably decreased in moderate and severe SARS-CoV-2 cases [15, 16]. Another proposed mechanism was described in a recent case-control study of 261 healthcare workers. The authors noted several prior studies that suggested both coronaviruses and influenza viruses engage with the angiotensin-converting enzyme 2 (ACE-2) and tetraspanin antibodies. Thus, there is belief that ACE-2 and tetraspanin antibodies may inhibit both coronavirus and low-pathogenic influenza A virus infections. Outcomes of this study pointed to a potential protective effect in those with influenza vaccination [11]. Additional studies reported that the influenza vaccine may lead to decreased risk of cardiovascular events due to potential interaction with immune and inflammatory systems to promote plaque stabilization [17, 18]. It has also been recently reported that influenza vaccine-induced antibodies may interact with the bradykinin 2 receptor, leading to an anti-inflammatory effect secondary to increasing nitric oxide [18, 19].

In a single-center study of 2,005 patients, Yang et al. were the first to perform a retrospective review highlighting a potential protective effect of influenza vaccination against adverse outcomes associated with SARS-CoV-2. Only 10.7% of patients in this study were considered up to date on their influenza immunization. The authors reported a 2.44 greater odds ratio (OR) for hospitalization and 3.29 greater OR for intensive care unit (ICU) admission indicating a protective effect for SARS-CoV-2 positive patients who were up to date on their influenza immunization [9].

This investigation seeks to explore the potential protective effects of influenza vaccination against SARS-CoV-2 using the TriNetX database. Specifically, this study aims to assess the possible benefit of influenza vaccination in mitigating critical adverse outcomes in SARS-CoV-2 positive patients using 73 million deidentified EMRs from 56 HCOs provided by a continuously updated network.

## Methods

**Fig 1:**
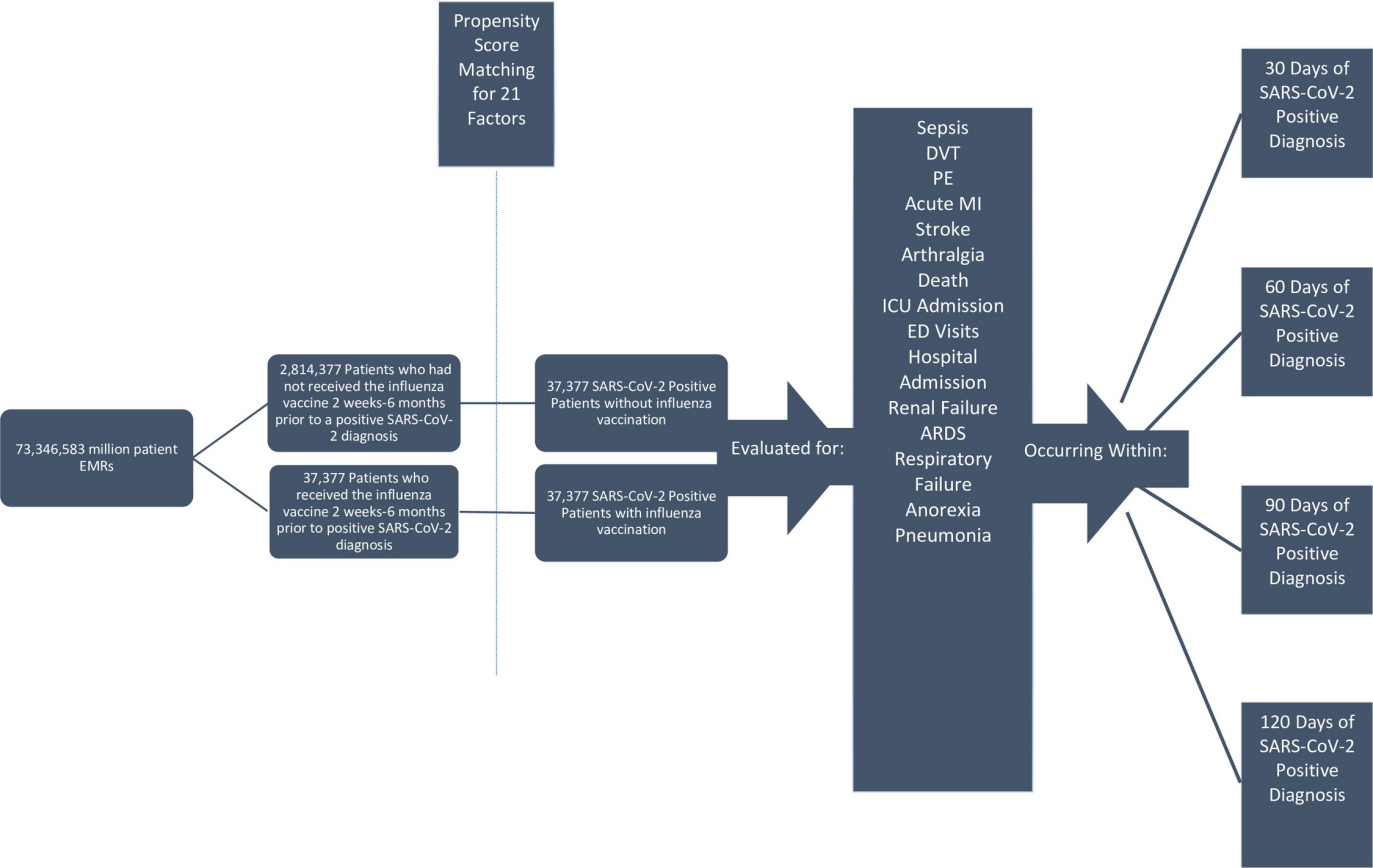
TriNetX search strategy that allowed the authors to narrow down the EMRs of an initial 73,346,583 patients into two propensity score matched 37,377-patient cohorts (N = 74,754) for direct comparison of adverse outcomes within 30, 60, 90, & 120 days of SARS-CoV-2-positive diagnosis.

At the time of our search in January 2021, the analytics subset contained EMRs from 56 HCOs distributed predominantly throughout the United States of America, but also with participating institutions in the United Kingdom, Italy, Germany, Israel, and Singapore. Within the US, the geographic distribution of HCOs is 6% in the Northwest, 33% in the Midwest, 42% in the South, and 19% in the West [3]. The deidentified records of 73,346,583 patients were retrospectively screened using the TriNetX platform (Fig 1).

In order to ensure accuracy, logical observation identifiers names and codes (LOINCs), the universal standard for identification of medical laboratory data, were used to identify patients positive for SARS-CoV-2 (LOINC 94500–6). CPT codes were used to identify patients who had received either the trivalent live intranasal (90660) or inactivated intramuscular influenza vaccine (90653) within a timeframe of six months–two weeks prior to receiving a SARS-CoV2-positive diagnosis. Additionally, Medicare patients receiving either the intranasal or intramuscular influenza vaccine were captured using the corresponding healthcare common procedure coding system (HCPCS) code (G0008). Any EMRs belonging to patients that were pregnant, incarcerated, experienced an outcome outside of a 120-day post-SARS-CoV-2 diagnosis window, or not meeting all of the aforementioned criteria by CPT code were excluded. Following application of inclusion and exclusion criteria, a cohort of 2,814,377 patients who had not received the influenza vaccine six months–two weeks prior to a positive SARS-CoV-2 diagnosis was compared to a second cohort of 37,377, patients who had received the influenza vaccine six months–two weeks prior to a positive SARS-CoV-2 diagnosis. We selected two weeks as the minimum end of our study’s timespan as it takes approximately two weeks for the immune system to fully develop antibodies following influenza vaccination. Conversely, six months was chosen as the maximum end of the timespan between influenza vaccination and SARS-CoV-2-positive diagnosis because the accepted standard for adequate protection without a waning effect is six months [20].

Following the creation of these two cohorts, we used the TriNetX platform to facilitate propensity score matching between cohorts with ICD-10 codes for numerous factors including age, race, gender, ethnicity, diabetes mellitus (E08-E13), elevated BMI status (E65-E68), hypertension (I10-I16), chronic ischemic heart disease (I25), heart failure (I50), COPD (J44), musculoskeletal disease (M00-M99), and factors influencing health status and contact with human services (Z00-Z99) which includes factors influencing health status including tobacco use, body mass index (BMI), and socioeconomic status. After propensity score matching, a cohort of 37,377 SARS-CoV-2 positive patients without influenza vaccination was paired with a second cohort of 37,377 SARS-CoV-2 positive patients, comparable in demographics and co-morbidities, that had received influenza vaccination within the aforementioned time frame.

Propensity score 1:1 balancing was completed within the TriNetX platform via logistic regression utilizing version 3.7 of Python Software Foundation’s *Scikit-Learn* package (Python Software Foundation, Delaware, USA). A greedy nearest neighbor matching algorithm approach was used, setting standard differences to a value of less than 0.1 to indicate appropriate matching. To eliminate record order bias, randomization of the record order in a covariate matrix occurs before matching. Baseline characteristics with a standardized mean difference between cohorts lower than 0.1 were considered well balanced.

Following optimization of the two cohorts for direct comparison, adverse outcomes were identified with ICD-10 or CPT codes as sepsis (A41.9), deep vein thrombosis (DVT) (I82.220, I82.40-I82.89, I82.A19), pulmonary embolism (I26), acute myocardial infarction (I21), stroke(I63), arthralgia(M25.5), ICU admission (99291, 1013729, 1014309), ED visits (1013711), hospital admission (1013659, 1013660, 1013699), renal failure (N19), acute respiratory distress syndrome (J80), acute respiratory failure (J96), anorexia (R63), pneumonia (J18), and death. Following identification, adverse outcomes within 30, 60, 90, and 120 days of SARS-CoV-2-positive diagnosis were analyzed and compared between the two cohorts. 120 days was made the maximum endpoint of our study window to account for the presence of the poorly understood Post-Acute Covid Syndrome (PACS), an autonomic dysfunction phenomenon observed in many patients after recovering from SARS-CoV-2 [17].

Using the TriNetX platform’s *Analytics* function, statistical analysis and logistical regression were performed by comparing indices and relative risks of outcomes following the successful matching of cohorts with a p-value greater than 0.05. Outcomes for all measures were calculated using 95% confidence intervals (CIs). All p-values were two-sided and the alpha level was set at 0.05. Risk ratio was defined in this study as the ratio of the probability of an adverse SARS-CoV-2-related event occurring without history of up-to-date influenza vaccination versus the probability of the same adverse SARS-CoV-2-related event occurring in a patient with history of up-to-date influenza vaccination [21].

Subsequently, Absolute Risk Reduction (ARR), defined as the difference in risk of an adverse SARS-CoV-2-related outcome between the influenza-vaccinated group and non-influenza-vaccinated group, was calculated for each adverse outcome. The reciprocal of ARR was then obtained to determine number needed to treat, henceforth referred to in this study as number needed to vaccinate (NNV), for all statistically significant variables. The NNV is a calculation specifying the average number of patients who needed to be up-to-date on their influenza vaccination in order to have prevented one adverse SARS-CoV-2-related outcome [22, 23].

## Results

Propensity score matching resulted in 37,377 patients in each cohort. Prior to matching, all between-groups factors were found to be significantly different (p<0.0001). However, following matching, all demographic and diagnostic factors were no longer significant (p>0·05) (Table 1), indicating successful balancing.

**Table 1:** Propensity score matching results, all factors were significantly different between the 2 cohorts prior to matching (p<0.0001).

				**Before Matching**					**After Matching**		
**Codes**	**Demographics**	**Mean +/-SD**	**Patients**	**% of Cohorts**	**p-Value**	**Std Dev**	**Mean +/- SD**	**Patients**	**% of Cohorts**	**p-Value**	**Std Dev**
AI	Age at Index	43.3 +/- 22.4 52.6+/-24.6	2,814,377 37,377	100% 100%	<0.0001	0.3959	52.5 +/- 24.6 52.6 +/- 24.6	37,377 37,377	100% 100%	0.7967	0.0019
2186–5	Not Hispanic or Latino		1,768,612 32,018	62.842% 85.662%	<0.0001	0.5406		32,085 32,018	85.842% 85.662%	0.4833	0.0051
2106–3	White		1,787,611 28,969	63.517% 77.505%	<0.0001	0.3104		29,037 28,969	77.687% 77.505%	0.5508	0.0044
F	Female		1,552,735 21,694	55.172% 58.041%	<0.0001	0.0579		21,696 21,694	58.046% 58.041%	0.9882	0.0001
M	Male		1,235,524 15,672	43.9% 41.93%	<0.0001	0.0398		15,666 15,672	41.913% 41.93%	0.9645	0.0003
2054–5	Black or African American		379,423 4,214	13.42% 11.274%	<0.0001	0.0671		4,204 4,214	11.248% 11.274%	0.9079	0.0008
UN	Unknown Ethnicity		776,781 3,127	27.6% 8.366%	<0.0001	0.5173		3,114 3,127	3.331% 8.366%	0.8635	0.0013
2131–1	Unknown Race		579,997 3,040	20.608% 8.133%	<0.0001	0.3614		3,054 3,040	8.171% 8.133%	0.8516	0.0014
2135–2	Hispanic or Latino		268,984 2,232	9.557% 5.972%	<0.0001	0.1343		2,178 2,232	5.827% 5.972%	0.4019	0.0061
2028–9	Asian		49,119 769	1.745% 2.057%	<0.0001	0.0229		736 769	1.969% 2.057%	0.3902	0.0063
1002–5	American Indian or Alaska Native		12,626 226	0.449% 0.605%	<0.0001	0.0216		209 226	0.559% 0.605%	0.4137	0.006
2076–8	Native Hawaiian or Other Pacific Islander		5,601 159	0.199% 0.425%	<0.0001	0.0406		137 159	0.367% 0.425%	0.2001	0.0094
**Codes**	**Diagnoses**	**Mean +/-SD**	**Patients**	**Percent of Cohorts**	**p-Value**	**Std Dev**	**Mean +/- SD**	**Patients**	**% of Cohorts**	**p-Value**	**Std Dev**
Z00-Z99	Factors Influencing Health Status & Contact with Health Services		1,231,075 37,146	43.742% 99.382%	<0.0001	1.5668		37,146 37,146	99.382% 99.382%	1.0000	<0.0001
M00-M99	Musculoskeletal Disease		691,394 22,362	24.567% 59.828%	<0.0001	0.7643		22,382 22,362	59.882% 59.828%	0.8814	0.0011
I10-I16	Hypertension		467,590 17,654	16.614% 47.232%	<0.0001	0.6953		17,679 17,654	47.299% 47.232%	0.8547	0.0013
E08-E13	Diabetes Mellitus		206,563 8,352	7.34% 22.345%	<0.0001	0.4318		8,348 8,352	22.335% 22.345%	0.972	0.0003
E65-E68	Overweight/Obesity		185,395 6,695	6.587% 17.912%	<0.0001	0.3507		6,619 6,695	17.709% 17.912%	0.4675	0.0053
I25	Heart Disease		122,995 5,409	4.37% 14.471%	<0.0001	0.3511		5,350 5,409	14.314% 14.471%	0.5387	0.0045
I50	Heart Failure		81,330 3,894	2.89% 10.418%	<0.0001	0.3056		3,849 3,894	10.298% 10.418%	0.5891	0.0040
J44	COPD		72,204 3,582	2.566% 9.583%	<0.0001	0.2970		3,539 3,582	9.468% 9.583%	0.5922	0.0039

Following matching, there were no significant differences between factors (p>0.05). (Black font = No Influenza Vaccine Cohort, Blue Font = Influenza Vaccine Cohort).

Following propensity score matching by the TriNetX system, statistical analysis was performed for all adverse outcomes of interest at 4 time points: 30, 60, 90, and 120 days following a SARS-CoV-2-positive diagnosis (Tables 2–5).

**Table 2:** Statistical analysis of adverse outcomes within 30 days of SARS-CoV-2-positive diagnosis.

	30 Days (N = 37,377)						
	No Vaccine	Vaccine	Risk Ratio	95% CI	p-value	Odds Ratio	95% CI
Sepsis	0.70%	0.52%	1.361	(1.123,1.650)	0.0016*	1.364	(1.124,1.655)
Stroke	0.27%	0.18%	1.479	(1.075,2.034)	0.0154*	1.480	(1.075,2.038)
ICU Admission	0.95%	0.80%	1.179	(1.003,1.385)	0.0457*	1.180	(1.003,1.389)
DVT	0.20%	0.15%	1.365	(0.959,1.944)	0.0829	1.366	(0.959,1.946)
PE	0.20%	0.21%	0.951	(0.69,1.31)	0.7565	0.950	(0.689,1.311)
Death	0.92%	1.04%	0.888	(0.768,1.025)	0.1054	0.887	(0.766,1.026)
ARF	1.01%	1.12%	0.901	(0.779,1.041)	0.1576	0.900	(0.777,1.042)
ARDS	0.14%	0.14%	1.019	(0.690,1.504)	0.9254	1.019	(0.690,1.505)
Arthralgia	0.95%	0.96%	0.996	(0.81,1.224)	0.9681	0.996	(0.809,1.226)
Renal Failure	0.09%	0.09%	0.996	(0.610,1.625)	0.9869	0.996	(0.610,1.626)
Anorexia	0.22%	0.22%	1.01	(0.740,1.379)	0.9496	1.010	(0.739,1.380)
Acute MI	0.35%	0.34%	1.047	(0.814,1.346)	0.7209	1.047	(0.813,1.348)
Hospital Admission	2.83%	2.67%	1.062	(0.960,1.176)	0.2417	1.064	(0.959,1.181)
ED Visit	1.35%	1.31%	1.034	(0.872,1.225)	0.7025	1.034	(0.871,1.228)
Pneumonia	0.77%	0.77%	0.996	(0.835,1.188)	0.9634	0.996	(0.833,1.190)

*Statistically Significant (p<0.05)

**Approaching Statistical Significance

**Table 3:** Statistical analysis of adverse outcomes within 60 days of SARS-CoV-2-positive diagnosis.

		60 Days (N = 37,377)					
	No Vaccine	Vaccine	Risk Ratio	95% CI	p-value	Odds Ratio	95% CI
Sepsis	0.91%	0.66%	1.383	(1.167,1.639)	0.0002*	1.386	(1.168,1.646)
Stroke	0.34%	0.23%	1.451	(1.096,1.92)	0.0089*	1.452	(1.096,1.924)
ICU Admission	1.14%	0.99%	1.156	(0.999,1.338)	0.0509**	1.158	(0.999,1.342)
DVT	0.29%	0.19%	1.531	(1.129,2.076)	0.0058*	1.532	(1.129,2.080)
PE	0.27%	0.28%	0.98	(0.741,1.295)	0.8848	0.980	(0.741,1.296)
Death	1.29%	1.42%	0.91	(0.805,1.029)	0.1328	0.909	(0.803,1.029)
ARF	1.24%	1.33%	0.933	(0.818,1.064)	0.3007	0.932	(0.816,1.065)
ARDS	0.16%	0.16%	1.016	(0.707,1.459)	0.9314	1.016	(0.707,1.460)
Arthralgia	1.55%	1.47%	1.049	(0.891,1.236)	0.5667	1.050	(0.889,1.240)
Renal Failure	0.11%	0.12%	0.925	(0.598,1.430)	0.7248	0.925	(0.598,1.430)
Anorexia	0.34%	0.29%	1.174	(0.905,1.523)	0.2258	1.175	(0.905,1.525)
Acute MI	0.46%	0.40%	1.146	(0.914,1.436)	0.2378	1.146	(0.914,1.438)
Hospital Admission	3.13%	2.91%	1.076	(0.977,1.185)	0.1367	1.079	(0.976,1.192)
ED Visit	1.82%	1.64%	1.108	(0.955,1.286)	0.1767	1.110	(0.954,1.291)
Pneumonia	0.94%	0.92%	1.017	(0.866,1.193)	0.841	1.017	(0.865,1.195)

*Statistically Significant (p<0.05)

**Approaching Statistical Significance

**Table 4:** Statistical analysis of adverse outcomes within 90 days of SARS-CoV-2-positive diagnosis.

		90 Days (N = 37,377)					
	No Vaccine	Vaccine	Risk Ratio	95% CI	p-value	Odds Ratio	95% CI
Sepsis	1.03%	0.71%	1.445	(1.229,1.699)	<0.0001*	1.450	(1.231,1.707)
Stroke	0.40%	0.27%	1.477	(1.141,1.912)	0.0029*	1.479	(1.141,1.916)
ICU Admission	1.25%	1.07%	1.174	(1.021,1.351)	0.024*	1.177	(1.022,1.355)
DVT	0.33%	0.23%	1.451	(1.096,1.920)	0.0089*	1.452	(1.096,1.924)
ED Visit	2.25%	1.87%	1.204	(1.050,1.380)	0.0076*	1.209	(1.052,1.390)
Anorexia	0.41%	0.32%	1.276	(1.001,1.627)	0.0486*	1.277	(1.001,1.630)
PE	0.31%	0.32%	0.99	(0.764,1.282)	0.9369	0.990	(0.763,1.283)
Death	1.53%	1.60%	0.957	(0.854,1.073)	0.4541	0.957	(0.852,1.074)
ARF	1.36%	1.42%	0.957	(0.844,1.086)	0.4979	0.957	(0.842,1.087)
ARDS	0.17%	0.17%	1.015	(0.715,1.441)	0.9339	1.015	(0.715,1.422)
Arthralgia	2.11%	1.78%	1.131	(0.977,1.309)	0.0998	1.133	(0.976,1.315)
Renal Failure	0.18%	0.12%	0.952	(0.627,1.445)	0.816	0.952	(0.626,1.446)
Acute MI	0.51%	0.45%	1.137	(0.917,1.409)	0.241	1.137	(0.917,1.411)
Hospital Admission	3.29%	3.04%	1.083	(0.985,1.190)	0.0975	1.086	(0.985,1.197)
Pneumonia	1.08%	1.01%	1.073	(0.922,1.248)	0.3634	1.073	(0.921,1.251)

*Statistically Significant (p<0.05)

**Approaching Statistical Significance

**Table 5:** Statistical analysis of adverse outcomes within 120 days of SARS-CoV-2-positive diagnosis.

		120 Days (N = 37,377)					
	No Vaccine	Vaccine	Risk Ratio	95% CI	p-value	Odds Ratio	95% CI
Sepsis	1.13%	0.78%	1.449	(1.240,1.690)	<0.0001*	1.454	(1.244,1.699)
Stroke	0.45%	0.29%	1.58	(1.233,2.026)	0.0003*	1.583	(1.234,2.031)
ICU Admission	1.37%	1.14%	1.202	(1.051,1.375)	0.0073*	1.205	(1.051,1.381)
DVT	0.36%	0.26%	1.411	(1.082,1.842)	0.0108*	1.413	(1.082,1.845)
ED Visit	2.58%	2.01%	1.285	(1.132,1.476)	0.0001*	1.293	(1.132,1.476)
Arthralgia	2.46%	2.02%	1.218	(1.064,1.395)	0.0041*	1.224	(1.066,1.405)
Hospital Admission	3.43%	3.15%	1.093	(0.997,1.200)	0.0587**	1.097	(0.997,1.207)
Anorexia	0.45%	0.36%	1.227	(0.975,1.545)	0.0808	1.228	(0.975,1.548)
PE	0.35%	0.33%	1.057	(0.822,1.360)	0.6646	1.057	(0.821,1.361)
Death	1.70%	1.67%	1.016	(0.910,1.133)	0.7827	1.016	(0.909,1.136)
ARF	1.49%	1.47%	1.014	(0.897,1.146)	0.8243	1.014	(0.896,1.148)
ARDS	0.18%	0.17%	1.095	(0.777,1.545)	0.6031	1.096	(0.776,1.546)
Renal Failure	0.14%	0.13%	1.037	(0.701,1.534)	0.8575	1.037	(0.700,1.535)
Acute MI	0.55%	0.47%	1.186	(0.963,1.460)	0.1075	1.187	(0.963,1.462)
Pneumonia	1.19%	1.05%	1.139	(0.984,1.319)	0.0805	1.141	(0.984,1.323)

*Statistically Significant (p<0.05)

**Approaching Statistical Significance

**Fig 2:**
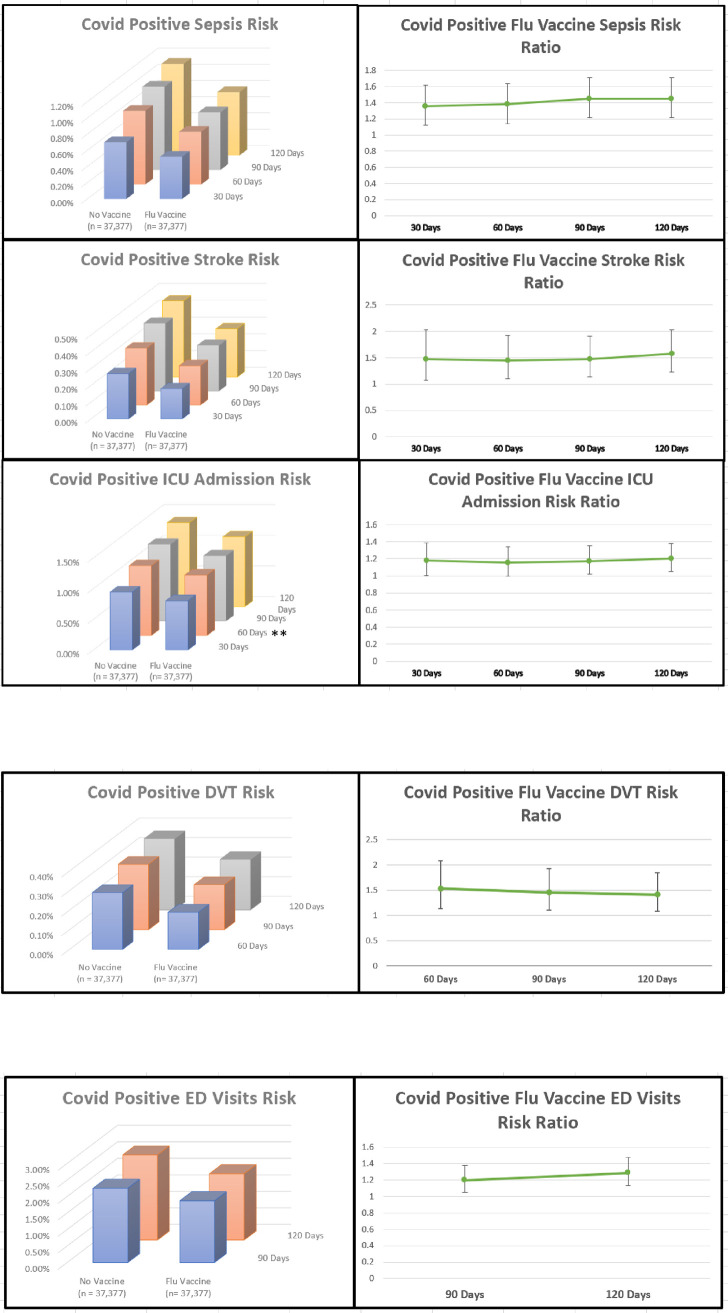
Significant adverse outcome trends 30–120 days (a), 60–120 days (b) & 90–120 days (c) (p<0.05). ** ICU Admissions Within 60 Days approaching significance (p = 0.0509, 95%).

SARS-CoV-2-positive patients who received the influenza vaccine experienced significantly decreased sepsis (p = 0.0001–0.0020, Risk Ratio: 1.361–1.450, 95% CI: 1.123–1.699) and stroke (p = 0.0003–0.0154, Risk Ratio: 1.451–1.580, 95% CI: 1.075–2.034) across all time points. ICU admissions were significantly lower in SARS-CoV-2-positive patients receiving the influenza vaccine at 30, 90, and 120 days (p = 0.0073–0.0240, Risk Ratio: 1.174–1.200, 95% CI: 1.003–1.385), while approaching significance at 60 days (p = 0.0509, Risk Ratio: 1.156, 95% CI: 0.999–1.338) (Fig 2A).

Patients who received influenza vaccination experienced significantly fewer DVTs 60–120 days after positive SARS-CoV-2 diagnosis (p = 0.0058–0.0108, Risk Ratio: 1.411–1.530, 95% CI: 1.082–2.076) (Fig 2B) and experienced significantly fewer ED visits 90–120 days post SARS-CoV-2-positive diagnosis (p = 0.0001–0.0076, Risk Ratio: 1.204–1.580, 95% CI: 1.050–1.476) (Fig 2C).

Additional findings included patients up-to-date on their influenza vaccination experiencing significantly less anorexia within 90 days of SARS-CoV-2-positive diagnosis (p = 0.0486, Risk Ratio: 1.276, 95% CI: 1.001–1.627) as well as decreased arthralgia within 120 days of SARS-CoV-2-positive diagnosis (p = 0.0041, Risk Ratio: 1.218, 95% CI: 1.064–1.395).

**Fig 3:**
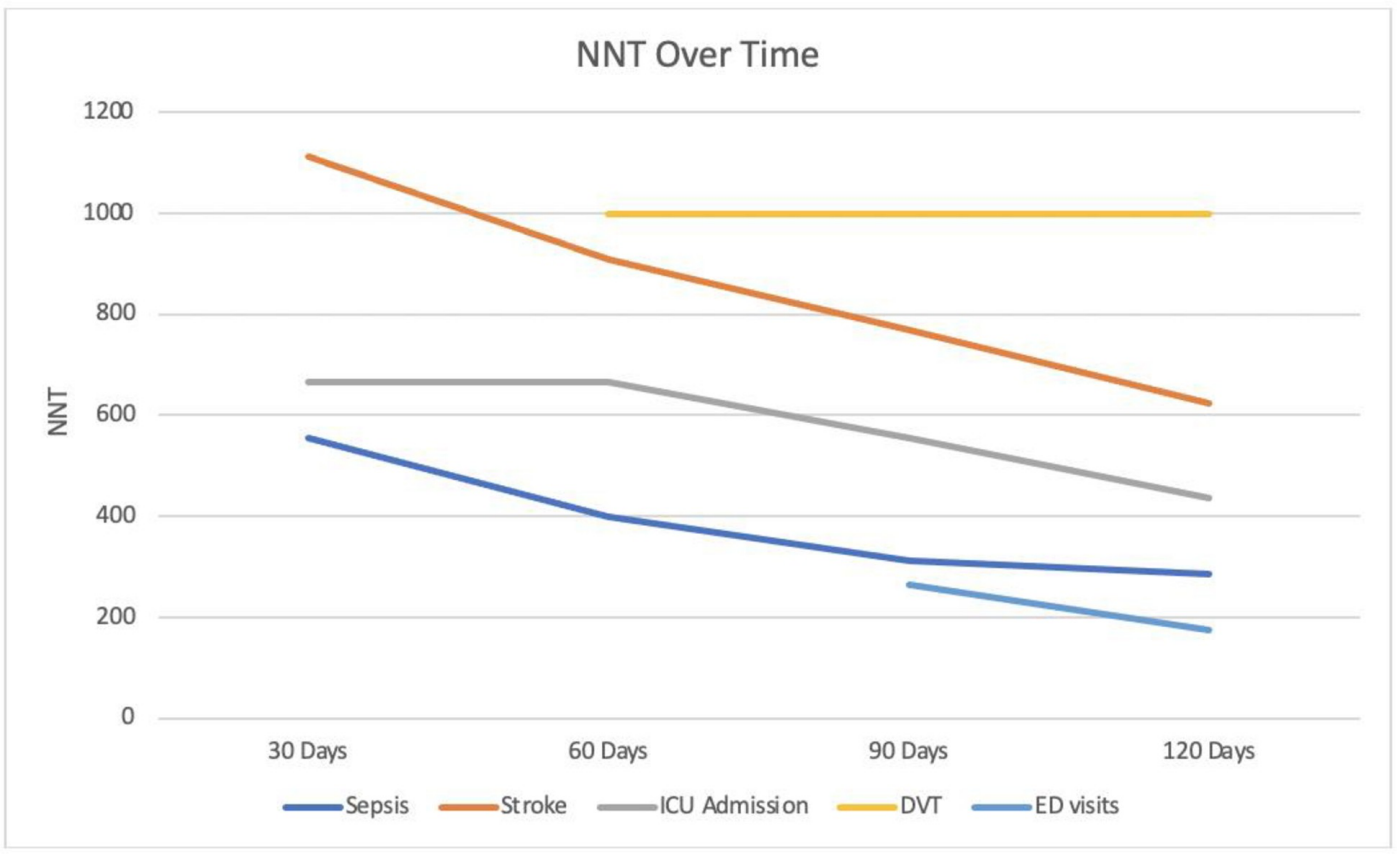
NNV to prevent adverse outcomes within 30–120 days, 60–120 days, and 90–120 days of SARS-CoV-2-positive diagnosis.

NNV with influenza vaccination to prevent one adverse SARS-CoV-2-related outcome calculations for significant findings for sepsis, stroke, and ICU Admission within 30, 60, 90, and 120 days of positive SARS-CoV-2 diagnosis are illustrated in Fig 3, along with NNV to prevent DVT within 60–120 days, and NNV to prevent ED Visits within 90–120 days (Fig 3).

## Discussion

This study underscores the utility of federated EMR networks as a potential solution for the need for urgent clinical data, particularly during health crises such as pandemics. While the work of retrospective single-center studies continues to have advantages such as detailed historical patient information that deidentified EMR networks cannot provide, the ability to scan, in minutes, the charts of 73 million patients from 56 HCOs in real-time to guide clinical decision-making is invaluable.

EMRs included in our study monitored patients with positive SARS-CoV-2 diagnoses for adverse outcomes during a period of 120 days. This time window was chosen intentionally to account for the possible presence of PACS. Although poorly understood, previous studies of PACS have reported orthostatic intolerance, often without objective hemodynamic abnormalities upon testing, as well as new illness-related fatigue to be the most common presentations. Development of these symptoms was found to occur between 0–122 days and 29–71 days post-SARS-CoV-2 diagnosis respectively [24, 25].

By focusing on rates of hospitalization and ICU admission, the study of Yang et al., garnered a sizable amount of media coverage [23, 24]. This study most closely mirrors this study’s aim of appraising the potential impact of influenza vaccination on adverse outcomes associated with SARS-CoV-2. Prior to comparing findings between these two studies, it is important to note several key differences in methodology [9]. While both studies relied on medical coding to identify SARS-CoV-2 positivity and influenza vaccination status, the timeframes were different, with this study encompassing the first full year of SARS-CoV-2 cases globally from January 2020-January 2021 [1, 25]. This timespan enabled our study to include data from the 2019–2020 influenza vaccine formulation as well as the most recent 2020–2021 influenza season formulation. This contrasts with the timespan of the previously mentioned study, as well as the recently published retrospective review of 27,000 patients by Conlon et al. Both of these studies analyzed SARS-CoV-2 cases between March-August 2020, a period overlapping between two different influenza vaccinations and seasons which excludes peak influenza season, and did not set a 2 week– 6 month time limit for influenza vaccine being “current/active” [9, 12]. Additionally, the Yang and Conlon study timeframes began 6 months after the CDC’s recommended influenza vaccination time in October, therefore the vaccine antibodies were likely already waning [9, 12, 20].

Our study found no association between influenza vaccination and risk of death in SARS-CoV-2-positive patients. This confirms the previous findings of Umasabor-Bubu et al., Pedote et al. and Ragni et al., which found that a history of influenza vaccination did not confer protection against death in reviews of 558, 664, and 17,600 patients respectively [26–28].

Alternatively, two macro-scale studies have found there to be conflicting relationships between influenza vaccination and mortality in the elderly population. In a large scale study of over 2,000 counties in the United States, Zanettini et al. demonstrated a potential protective effect of influenza vaccination on SARS-CoV-2 mortality [29]. Conversely, Wehenkel et al. performed a macro-scale study of association between influenza vaccination rate and SARS-CoV-2 deaths in an examination of over 500,000 patients across 39 countries [30]. This study showed a positive association between COVID-19 deaths and influenza vaccination rates in elderly people 65 years of age and older. The conflicting findings of these studies may be attributable to their large scale nature and lack of analysis of individual patient EMRs, thereby further increasing the need for prospective randomized control studies to better define the potential protective effect of influenza vaccination against SARS-CoV-2.

In light of the over 140 million confirmed positive cases worldwide^1^, the use of NNV calculations allows for a deeper appreciation of the potential benefit of influenza vaccination. In addition to guarding against a possible “twindemic” of simultaneous outbreaks of influenza and SARS-CoV-2 [31], the NNV trends observed within 30–120 days of SARS-CoV-2 diagnosis for sepsis, stroke, ICU admission, DVT, and ED visits further strengthen the case in favor of a protective effect of influenza vaccination (Fig 3). Specifically, in order to prevent one individual from visiting the ED, developing sepsis, being admitted to the ICU, suffering a stroke, or having a DVT within 120 days of positive SARS-CoV-2 diagnosis, 176, 286, 435, 625, and 1,000 people respectively would need to have been up-to-date with their influenza vaccination. When considered on a global scale, the NNVs calculated in this study may serve to benefit not only those that will be infected with SARS-CoV-2, a diagnosis that has already affected over 140 million to date, but also the finances and resources of the health systems responsible should patients suffer these adverse outcomes [32]. Even more encouraging, apart from DVT for which NNV remained stable, the NNVs for sepsis, stroke, ICU Admissions, and ED Visits were down trending at the 120-day mark, implying that the NNV and thus potential protective benefit of influenza vaccination may be even stronger than observed in the present study.

Expanding upon our prospective understanding of the relationship between influenza vaccination and protection against adverse outcomes during SARS-CoV-2 is the work of Pawlowski et al. This retrospective review found that a history of eight different vaccines including Polio, H. influenzae type-B, measles-mumps-rubella, and Varicella, amongst others, within the past one, two, or five years is associated with decreased SARS-CoV-2 infection rates, even after cohort balancing [33]. This suggests that the protective effect observed by our group and others against SARS-CoV-2 may not be unique to influenza vaccination.

This study has the benefits of large cohort size and a tightly matched patient population, however reliance on a global database also introduces limitations that must be acknowledged. These limitations include our study’s retrospective nature, absence of detailed historical patient data, and lack of ability to follow up regarding new symptoms. Our search query’s reliance on the CPT, ICD-10, and LOINC coding of individual HCOs is another potential source of confounding as the accuracy of these factors is inherent to the EMRs comprising the database. This statement is particularly of interest as relates to false positive and false negative cases of SARS-CoV-2, which relies on the specificity and sensitivity of PCR and rapid antigen testing.

Federated EMR networks, such as TriNetX, have vast potential to challenge or verify scientific findings using sample sizes and turnaround times unachievable by individual centers, particularly during health crises such as pandemics. Our study was able to verify and challenge the relatively large difference in the potential protective effect of influenza vaccination observed by the previous study with a much more modest effect backed by nearly 75,000 global EMRs [9]. The potential protective effects of the vaccine against sepsis, stroke, DVT, ED visits, and ICU admissions at 30, 60, 90, and 120 days following SARS-CoV-2-positive diagnosis reaffirms the importance of annual influenza immunization.

While this observed potential protective effect is relatively small, the stringently matched cohort balancing and sample size afforded by TriNetX substantially increases our confidence in the fidelity of our findings. In the context of over 140 million cases globally, the potential protective benefits further elucidated by the NNV calculations for these same adverse outcomes suggests that a concerted effort to continue ramping up influenza vaccination in parallel with SARS-CoV-2 vaccination is strongly worth consideration. Although production and distribution of SARS-CoV-2 vaccines continues to increase daily, the fact remains that certain populations in the global community may still have to wait a long period of time before they are vaccinated and could therefore benefit from a more readily available source of even marginally increased protection [34]. That being said, less than half of US adults receive influenza vaccination each year, with Non-Hispanic Black, Hispanic, and American Indian/Alaskan Native individuals having had the lowest influenza vaccination coverage while also being disproportionately affected by SARS-CoV-2 [35].

The influenza vaccine may be a viable option to attenuate the adverse effects of SARS-CoV-2 worldwide, with a specific potential to benefit populations struggling with access to or distribution of SARS-CoV-2 vaccination. Even patients who have already received SARS-CoV-2 vaccination may stand to benefit given that the SARS-CoV-2 vaccine does not convey complete immunity, although further research into the relationship and potential interaction between influenza vaccination and SARS-CoV-2 vaccination should be performed.

## Conclusion

Using a federated EMR network of over 73 million patients across 56 global HCOs, this analysis examines the potential protective effect of the influenza vaccine against various adverse outcomes at 30, 60, 90, and 120 days of SARS-CoV-2-positive diagnosis. Significant findings in favor of the influenza vaccine in mitigating the risks of sepsis, stroke, DVT, ED visits, and ICU admissions suggest a protective effect that merits further investigation. Limitations include this study’s retrospective nature and its reliance on the accuracy of medical coding. Future prospective controlled studies to validate these findings and determine if an increased emphasis on influenza vaccination will improve adverse outcomes in SARS-CoV-2-positive patients will be beneficial.
